# Condition-dependent survival and movement behavior in an endangered endemic damselfly

**DOI:** 10.1038/s41598-023-48162-w

**Published:** 2023-12-09

**Authors:** Hayat Mahdjoub, Rabah Zebsa, Amin Kahalerras, Hichem Amari, Soufyane Bensouilah, Michael J. Samways, Rassim Khelifa

**Affiliations:** 1 Biology Department, Concordia University, 7141 Sherbrooke St. W., Montreal, QC H4B 1R6 Canada; 2 Department of Nature and Life Sciences, Faculty of Nature and Life Sciences and Earth and Universe Sciences, University of 08 May 1945, Guelma, Algeria; 3 Direction Générale Des Forêts, 24000 Guelma, Algeria; 4 Department of Natural Sciences, Ecole Normale Supérieure de Ouargla, Ouargla, Algeria; 5 Biology Department, Université de Laghouat, Laghouat, Algeria; 6 Department of Conservation Ecology and Entomology, Stellenbosch University, Stellenbosch, South Africa; 7 Institute for Resources, Environment, and Sustainability, University of British Columbia, 2202 Main Mall, Vancouver, BC V6T 1Z4 Canada

**Keywords:** Biodiversity, Conservation biology

## Abstract

Movement is essential for the maintenance of populations in their natural habitats, particularly for threatened species living in fluctuating environments. Empirical evidence suggests that the probability and distance of movement in territorial species are context-dependent, often depending on population density and sex. Here, we investigate the movement behavior of the spring cohort of an endangered endemic damselfly *Calopteryx exul* in a lotic habitat of Northeast Algeria using capture-mark-recapture (CMR) of adults. By sampling 10 gridded river stretches across a 2 km section of the watercourse, we were able to estimate the distance of movement throughout individual lifespans and estimate movement probability for both males and females. We used multistate models to examine whether individual density and sex ratio influence survival and movement probability. We found that males and females had similar movement kernels with most individuals moving short distances (83% performing movements of < 100 m and only 1% > 1000 m). Of the 547 marked individuals, 63% were residents, and 37% were movers (moved at least 50 m from one sampling occasion to another). Survival probability showed higher estimates for females and was slightly density-dependent (i.e., lower survival probabilities were associated with high male densities). Survival probability did not show a marked difference between residents and movers. Movement probability and distances were positively correlated with individual density, but were not or slightly correlated with sex ratio, respectively. These results are not in line with the hypotheses of sex-biased movement and survival costs of movement. Our results suggest that the species performs mostly short-distance movements that are dependent on intraspecific interactions.

## Introduction

To stay or to move is a common dilemma that animals face during their lives. Understanding the determinants of movement strategies of species and their intraspecific variability have been central topics in the field of ecology and evolution. Movement plays a crucial role in various ecological and evolutionary processes^1–3^. Theoretical studies suggest that individual movement such as dispersal is essential for maintaining viable populations in variable environmental and social conditions^4^. Empirical studies have shown that movement is often condition-dependent, that is, it is triggered by factors such as habitat degradation, competition, and predation^5,6^. In fragmented habitats, or habitat with unpredictable ecological conditions (e.g., climate fluctuations, anthropogenic stress), movement offers several benefits, such as promoting gene flow, facilitating colonization of new habitats, and reducing competition for resources^7,8^. However, movement also comes with costs, including energy expenditure and increased vulnerability to predation^9^. For species of conservation concern, understanding factors that drive movement behavior and capacity is critical for implementing effective conservation plans^10^.

While animal movement has been investigated extensively in taxa such as birds and mammals, there has been a growing interest among researchers in using insects^11,12^. Odonates, particularly damselflies, have been frequently used in the study of the ecology of movement and dispersal using a wide range of experimental and observational methods^13–15^. They are suitable organisms for the study of drivers of movement because they are easy to mark and recapture, measure in the field, and survey throughout their lifespan^16^. While long-range movements occasionally occur, most movements are short-range movements (< 100 m)^17–19^. Studies have shown that damselflies have female-biased dispersal similar to birds and mammals^15^. In territorial species such as Calopterygidae^20^, where a large number of females is monopolized by a few dominant males, movement could be density-dependent and sex-biased^20,21^. For males, male-male competition may result in density-dependent movement to new suitable patches with less frequent costly interactions^22^. For females, a large number of ovipositing conspecific females could be used as a social cue for habitat suitability^23–25^. An absence of both density-dependent and sex-biased movement is rare and has not been recorded in this group.

*Calopteryx exul* is an endemic damselfly resident in Morocco, Algeria, and Tunisia. Current records on the geographic range of the species suggest a relatively fragmented range^26^. The species is among the few odonates currently listed Endangered in the North African IUCN Red list. The species inhabits lotic systems with relatively fast-flowing water, a habitat type that has experienced degradation over the last few decades in Algeria, leading to several populations being extirpated^27^. Due to its rarity and the low number of surveys, the species remained unrecorded for nearly a century (from 1910 to 2007) in Algeria^28^. While new populations were recorded during the decade after its discovery in the Seybouse river^24,25^ several populations have either perished or suffered severe degradation of their habitat^31^.

*C. exul* has a long flight season with two cohorts^32^; the first is larger and longer (hereafter spring cohort), spanning May to July, whereas the second is smaller and shorter, spanning mainly September to October (hereafter autumn cohort). Like other Calopterygidae species^33–35^, it is territorial and males guard patches of floating leaves where females lay eggs after copulating with the territorial male^36^. One record of 5 km movement suggests that the species is able to perform relatively long-distance movements^18^. However, apart from this record, the movement behavior of the species has never been studied.

Here, we investigate the movement behavior of the spring cohort of *C. exul* between patches of a lotic habitat using capture-mark-recapture of adults along a 2 km-stretch of the Seybouse river (Northeast Algeria). We test whether population density and sex ratio influence the probability of survival and movement behavior using multistate models. We also assessed the distances of movement of adults across the river and their potential correlation with population density and sex ratio. We hypothesize that larger densities and male-biased sex ratios reduce survival probability and increase the probability of movement.

## Material and methods

### Study site

**Figure 1:**
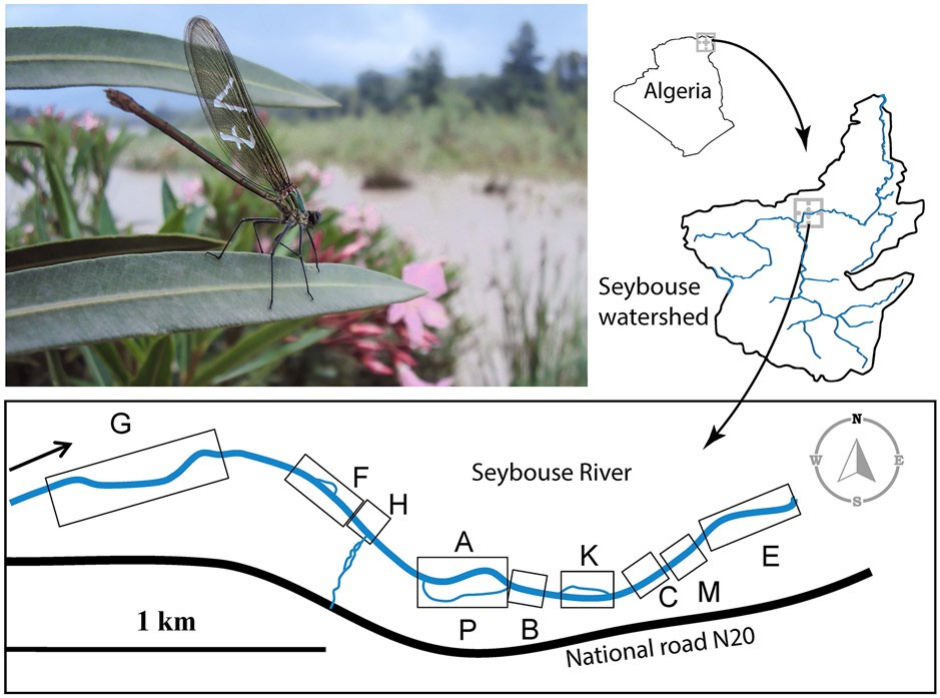
Geographic location of the study site with a representation of spatial distribution of all 10 river sections sampled. The picture is a female *Calopteryx exul* in the study site. Details about the 10 river sections are given in Table S1.

The study was conducted in the Seybouse river, located in the northeastern part of Algeria (Fig. 1). The local climate has a Mediterranean climate (Csa Köppen climate classification) characterized by hot and dry summers and cool and wet winters. The Seybouse river has an average annual rainfall ranging from 350 mm upstream to 608 mm downstream. The hydrology of the area has a wet season from October to May, followed by a dry season lasting from June to September. The behavioral investigation took place upstream of the Seybouse River, about 5 km west of Guelma city (36°28,023.16″N and 7°22,032.73″E), where elevation was 210 m above sea level. One of the largest populations of *C. exul* occupied this location in 2011^37^. The watercourse is a shallow stream, width of 2–4 m. The vegetation along the banks is predominantly *Typha angustifolia* L., *Cyperus longus* L., *Juncus maritimus* Lam., and *Paspalum distichum* L. In this region, the species coexists with other lotic species such as *C. haemorrhoidalis* Vander Linden, *Platycnemis subdilatata* Selys, and *Gomphus lucasii* Selys.

### Capture-mark-recapture protocol

We carried out capture-mark-recapture sampling on a daily basis at the study site on the first (spring) cohort of the flight season from 4 to 25 May 2011, starting from 9:30 h and ending at 16:00 h. Every day during the study period, six field workers conducted adult captures along 10 accessible river sections distributed along a 2 km river reach. The length of each river section varied between 80 and 235 m (135 ± 47.3 m, N = 10) (Table S1). The variation in the lengths of river segments resulted from the presence of dense vegetation along the riverbanks, which made some sections of the river inaccessible (mostly *T. gallica* trees). Each river section was divided into 10 m-stretches using flags to estimate the exact location within the stretch and to assess individual movement as well as the distance of movements from one sampling occasion to another. We used hand nets to capture adults and made sure that individuals were handled with care. All participants who sampled the species had prior experience in capturing and handling odonates. We were not able to estimate the age of individuals because of the lack of age-dependent traits in the species (e.g., change in colors). Tenerals and young imagos (individuals with soft wings and body) were not included in this study. Also, individuals with extensive wing wear (which reflects old age) were not recorded in our study. Based on the rigidity of the wings and behavior (e.g., territoriality, mating behavior), we assume that all marked individuals were mature. To mark captured adults, we wrote an alphanumeric code on the hindwing using permanent markers (Edding paint marker 780). Individuals were released at the same location of capture by gently placing them on plant leaves of the bank vegetation. During our daily CMR sampling, we recorded the code, the sex, and the location of individuals. For each marked or recaptured individual, we also calculated individual density and sex ratio within 10 m radius (based on the sample of marked and recaptured individuals) to determine their correlation with survival and movement behavior.

### Capture-mark-recapture modeling

Multistate models (MS) were used to estimate recapture, survival, and transition probabilities^38^. MS models, an extension of Cormack Jolly Seber (CJS) models, allow for modeling not only survival but also transitions between individual states. A state represents a time-specific characterization of an individual, such as site, reproductive state, or physical condition. Unlike CJS models where encounter history is denoted by ‘0’s (not detected) and ‘1’s (detected), MS models specify different states of detection (1s), except for the initial release. In this study, we use two states: resident (denoted as 1) and mover (denoted as 2). The latter is assigned where individuals move more than a certain distance threshold away from their previous encounter, whereas a resident was an individual that stayed within the distance threshold of the previous record. We used three distance thresholds: 50 m, 100 m, and 500 m, which based on previous studies on congeneric species correspond to common, less common, and relatively rare movement distances^21^. Based on these distance thresholds, we produced three encounter histories. An encounter history consists of ‘0’s, ‘1’s, and ‘2’s, representing absence (not detected), residence (distance ≤ threshold), and non-resident (distance > threshold), respectively. For example, an encounter history of ‘1012’ indicates that the individual was marked and released on day 1 in patch A, not recaptured on day 2, observed again in patch A on day 3, and detected away from patch A at a distance that exceeded the distance threshold (50 m, 100 m, or 500 m) on day 4. The parameters estimated by MS models are (1) survival probability (S), which represents the probability of surviving from occasion i to i + 1 given that the animal was philopatric or non-philopatric, (2) recapture probability (p), which is the probability of encountering an individual conditional on being alive in a particular state, and (3) transition probability (Psi), the probability of transitioning from one state to another, given the survival of individual from i to i + 1.

Candidate models were selected to analyze p, S, and Psi. Initially, we set S and Psi constant and varied p with time (continuous and categorical), sex, and state (resident or mover). We built the list of models starting from the simplest one (S(.) p(.) Psi(.)) to the most complex one incorporating all covariates (S(.) p(time + sex + state) Psi(.)). Model selection was then conducted using the corrected Akaike information criterion (AICc). Once we determined the best model for detection probability, we repeated the same process of model selection for survival probability by fixing the best model for p and testing for the effect of sex, time, state, individual density (average across the lifespan), and sex ratio (average across the lifespan) on S. We selected the best model for S, and repeated the model selection process for transition probability (movement probability) by testing for the effect of state, sex, individual density, and sex ratio. Variance inflation factor (c-hat), which is an estimate of the fit of the CMR model to the observed data, was calculated by dividing the overall χ^2^ (sum of the TEST2 and TEST3 component tests) by the overall degrees of freedom^39^. As the c-hat value was ≈ 1 (c-hat = 0.95), no variance adjustment was applied to the models.

### Statistical analyses

We used R 4.2.2 to perform our statistical analyses^40^. We used a chi-squared test to determine whether sex ratio of our sample deviated from unity. We also used chi-squared tests to determine whether there was a sex bias in the proportion of individuals recaptured at least once, the proportion of movers, and the direction of movement (upstream or downstream). All mixed-effects models were conducted with lme4^41^. We tested whether the density of males and females around focal individuals deviated from a slope of 1 using a generalized mixed-effects model (with a Poisson error distribution). This model included male density as a response variable, female density (including the offset() function which is typically used to adjust the linear predictor for a specific variable) and sex as explanatory variables, and individuals’ identification (ID) as a random effect. Here, the offset function is used to determine whether the slope of male-to-female density deviates from 1.

We also tested the relationship between individual density around focal individuals and sex ratio using a generalized mixed-effects model that includes individual density as a response variable, the quadratic term of sex ratio and sex as explanatory variables, and individual ID as a random effect. The test of goodness of fit on the MS model was conducted using the function *release.gof* from the RMark package^42^. These tests assess whether all marked individuals have equal chances of being captured (Test2) and whether they have an equal probability of survival (Test3) on any one occasion^43^. The parameter estimates for p, S, and Psi were obtained using the R package RMark. Biologically meaningful candidate models were selected for the three parameters. Models with ΔAICc < 2 were considered, and the most parsimonious model was selected as the best one. We tested for the difference in the shape of the movement kernel of males and females using a two-sample Kolmogorov–Smirnov test. To test whether individual density and sex ratio were correlated with movement distance, we computed a generalized mixed effects model with Poisson error, including distance as a response variable, individual density, sex ratio, and sex as explanatory variables, and individual ID and observation ID (to account for overdispersion^44^) as random effects.

## Results

Our study included a total of 547 marked individuals, 255 males and 292 females. The sex ratio (53.4% females) did not show a significant difference from unity, both when considering all individuals (χ^2^ = 2.50, *P* = 0.11) and when considering only individuals recaptured at least once (χ^2^ = 2.96, *P* = 0.08). Among the recaptured individuals, 55.5% were recaptured at least once (57.1% in males and 53.7% in females). Of these, 63% were resident (did not move more than 50 m), while 37% were movers (moved at least 50 m). This difference in the proportion of mover and resident individuals was significant (χ^2^ = 34.31, *P* < 0.0001). The direction of movement (at least 50 m) was not biased towards upstream or downstream in both males (χ^2^ = 3.11, *P* = 0.07) and females (χ^2^ = 1.37, *P* = 0.24).

**Figure 2:**
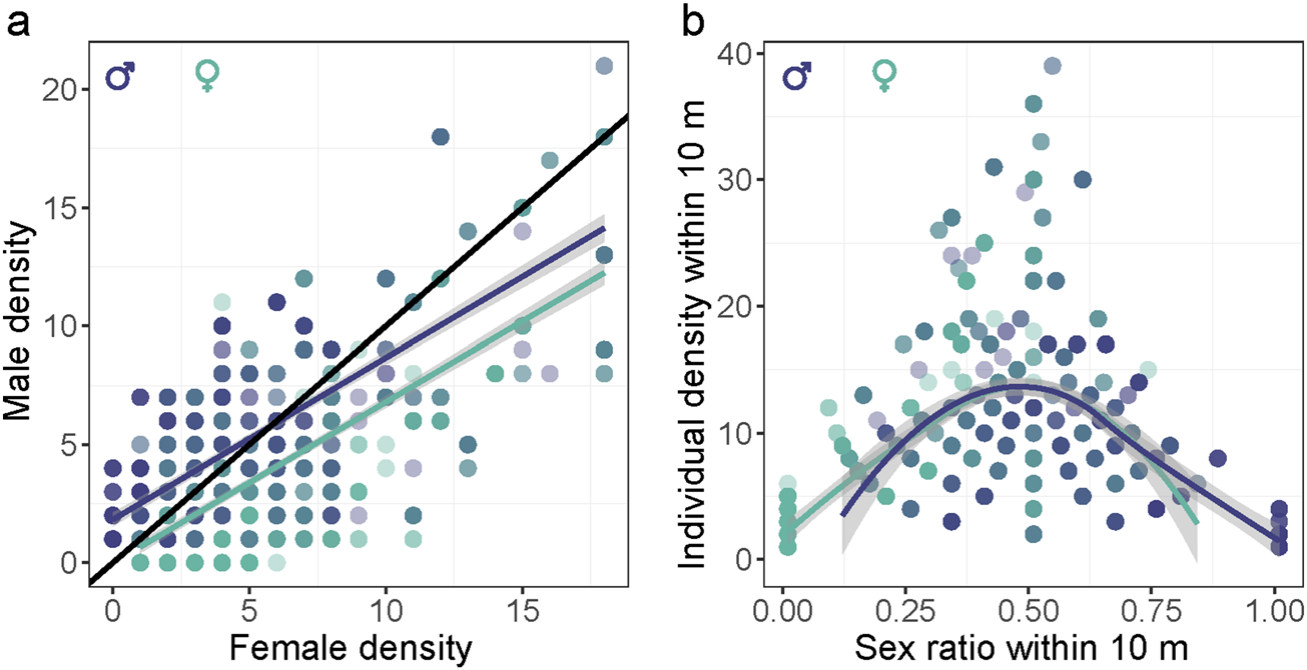
(**a**) Correlation between male and female densities (within 10 m radius) surrounding our studied males and females. The diagonal line is a reference line with a slope of 1 and an intercept of 0. Regression lines are simple linear regressions for focal males and females. (**b**) Relationship between individual density (males + females) and sex ratio surrounding (within 10 m radius) our focal males and females. Curves are loess regressions for males and females.

Male and female densities were positively correlated, but the slope deviated from equality (1:1) such that high female densities were associated with lower male densities (male density: z = − 313.10; *P* < 0.0001) (Fig. 2a). Overall, higher densities of individuals were more likely to have an even sex ratio, as revealed by the quadratic effect of sex ratio (sex ratio: z = 36.35; *P* < 0.0001; sex ratio^2^: *t* = − 36.74; *P* < 0.0001) (Fig. 2b).

### Recapture, survival, and movement probabilities

**Figure 3:**
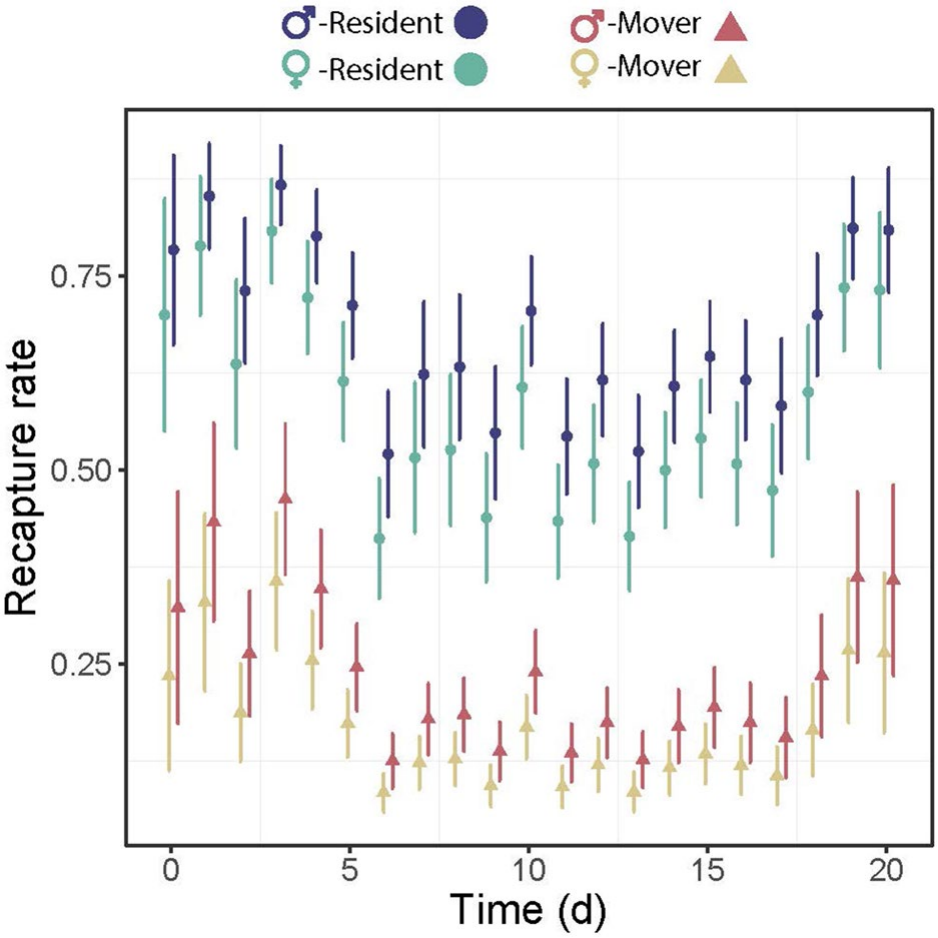
Recapture rate of resident and mover (movement of ≥ 50 m) males and females throughout the sampling occasions in *Calopteryx exul*. Error bars are confidence intervals.

The goodness-of-fit tests did not yield significant results, indicating that there is no deviation from the assumptions necessary for applying the multistate model to the three types of encounter histories (Table S2). The selection of the best model for detection probability revealed that it included the additive effects of time, movement state, and sex (Tables S3–S5). A large difference in recapture rates was found between residents and movers with the former being 48% more likely to be resighted than the latter (Fig. 3). The difference in recapture probability between males and females was relatively small, with males exhibiting (on average) 8% higher recapture rate compared to females.

**Figure 4:**
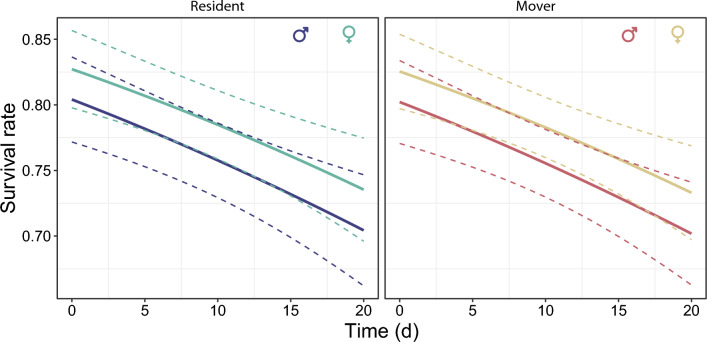
Survival probability of resident and mover males and females of *Calopteryx exul* throughout the season. Error bars are 95% confidence intervals.

**Figure 5:**
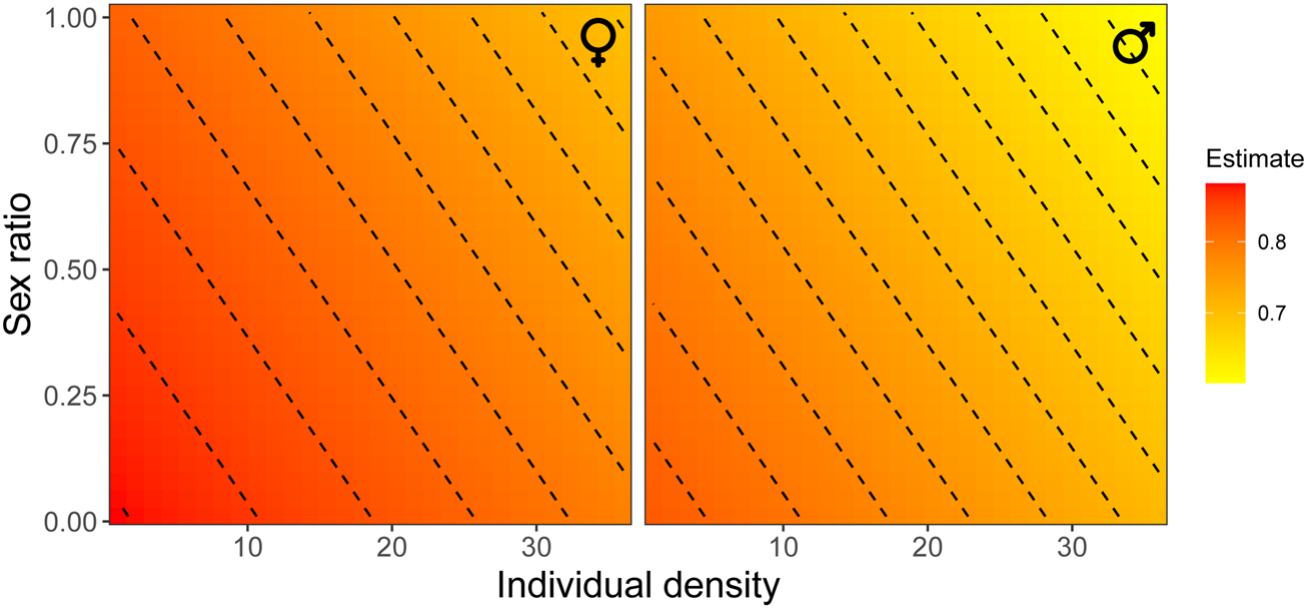
Survival probability of *Calopteryx exul* males and females depending on individual density and sex ratio.

There were three to four top models for survival probability that had ΔAICc < 2 (Tables S3–S5). The most parsimonious model includes only sex, regardless of the distance threshold of movement (50 m, 100 m, or 500 m). However, more than half of the top model for survival probability included the additive effects of movement state and individual density. At least one of the top models included sex, sex ratio, and time. Females had on average a slightly higher survival probability than males (Fig. 4), with 0.80 [95% CI 0.77–0.82] in females and 0.75 [95% CI 0.72–0.78] in males. The model that includes time predicts that survival probability showed a temporal decline (Fig. 4). Interestingly, residents and movers had relatively similar survival probabilities regardless of the distance threshold of movement. Top models that included individual density and sex ratio predicted that survival probability was highest when individual density was low and the sex ratio was female-biased, and lowest when individual density was high a sex ratio was male-biased (Fig. 5).

**Figure 6:**
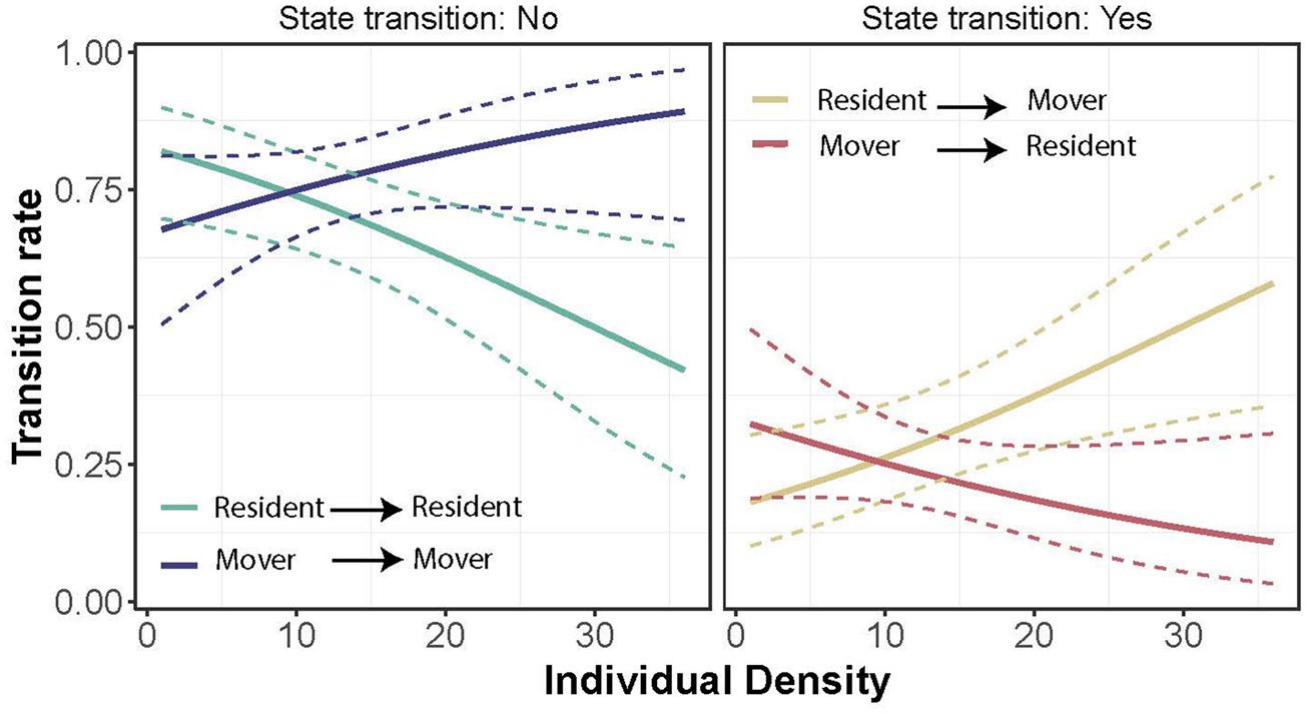
Transition probability of male and female *Calopteryx exul* depending on individual density surrounding individuals.

The top model for transition probability included movement state and individual density (Table S3–S5). MS models for transition probability using movement thresholds of 50 m and 100 m showed a single model with ΔAICc < 2. MS models using a movement threshold of 500 m showed two models with ΔAICc < 2, with the most parsimonious being constant (Table S5). Our model predicted that the probability of remaining in the same state was higher than transitioning to a new state, that is, residents were more likely to remain residents and movers were more likely to remain movers than to shift states. Interestingly, the probability of remaining a resident was negatively correlated with individual density, whereas the probability of remaining a mover was positively correlated with individual density (Fig. 6). The mean transition probabilities from resident to mover was 0.27 (95% CI = 0.19–0.36) and from mover to resident was 0.24 (95% CI = 0.17–0.32). Transition probabilities from resident to mover declined with individual density, whereas that from mover to resident increased with individual density.

### Movement distance

**Figure 7:**
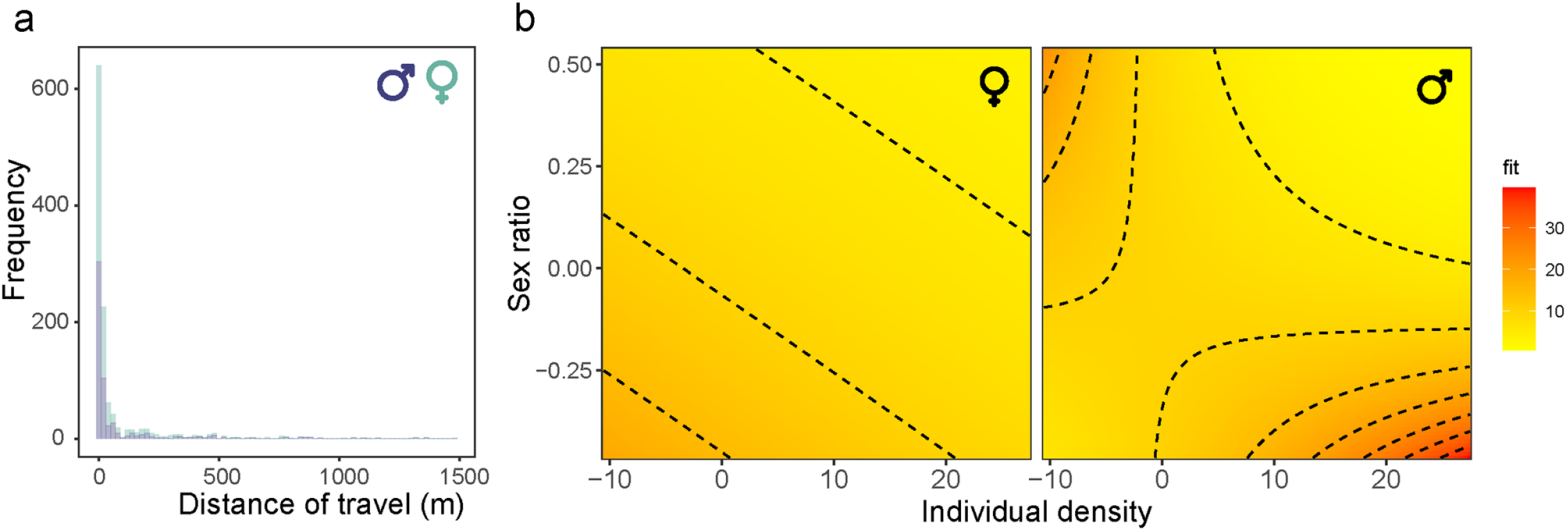
(**a**) Movement distances of male and female adults of *Calopteryx exul*. (**b**) Movement distances relative to individual density and sex ratio in *C. exul*. Sex ratio was calculated as the number of males divided by the total number of individuals.

The movement kernel of males and females were similar (Kolmogorov–Smirnov test: D = 0.03, *P* = 0.93) (Fig. 7a), showing a median of 8 m and 9 m and a mean of 76.2 ± 186 m (max = 1358 m; n = 583) and 77.2 ± 187 m (max = 1475 m; n = 675), respectively. Movement kernels were highly right-skewed, with 17% of distances > 100 m, 4% > 500 m, and 1% > 1000 m. Movement distance was correlated with individual density (χ^2^ = 8.64, *P* = 0.003) and the interaction of sex and sex ratio (χ^2^ = 5.37, *P* = 0.02). These results indicate that males that were recorded in areas with high female densities travelled the longest distances (Fig. 7b).

## Discussion

We examined the movement behavior of the spring cohort of an Endangered damselfly in North Africa using capture-mark-recapture to understand whether individual density and sex ratio influence the probability and distance of movement of males and females. We found that (1) both males and females showed similar movement tendency albeit with rare movements ≥ 1 km, (2) survival probability was influenced mainly by sex but also movement, individual density and sex ratio, (3) movement probability was not influenced by sex but was density-dependent, and (4) movement distance was correlated with density of individuals and sex ratio (interacting with sex). The study suggests that movement was local and highly dependent on the social context. Our findings add new perspectives on condition-dependent movement in regions with unpredictable climates such as the Mediterranean basin^45^ for a threatened species that has not been well-studied.

Contrary to the prediction that there is sex bias in the movement of territorial species (mostly female-biased^15^), our study shows that males and females had a similar probability of movement. The overall movement behavior, including direction (upstream or downstream), distance, and probability was similar in both sexes even though the recapture rate was slightly higher in males than females. Male-biased recapture probability is widespread in odonates^16^, and is mostly due to female cryptic behavior. A previous CMR study on *C. exul* in the same region also showed higher recapture rates for males (0.56 vs. 0.29)^46^. In other odonates, male-biased recapture probability was recorded in different species such as *Calopteryx haemorrhoidalis* (0.831 vs. 0.786^47^), *C. splendens* Harris (0.487 vs. 0.271^48^), *C. maculata* Palisot de Beauvois (0.660 vs. 0.494^49^), and *Ischnura gemina* Kennedy (0.807 vs. 0.540^50^). It is likely that the similarity in movement behavior between sexes is due to individuals seeking similar resources such as territories (patches of vegetation to mate), and that individuals were recorded to travel various distances (from a few meters to more than 1 km) to reach them. Most movements did not exceed 100 m, which is typical in damselflies^19^, including Calopterygidae^21^. In a closely related European species (*C. splendens*), Chaput-Bardy, et al.^21^ showed that females dispersed more often than males, although the distances travelled were similar. It is likely that the low availability of preferred plant patches for *C.exul* reduced the probability of movement for females. Mellal, et al.^46^ showed in a field experiment that providing additional plant patches promoted female and male colonization, and increased recapture rate (suggesting a reduction in movement probability). Similar to studies on other damselfly species^17,21,51^, our results on movement behavior suggest that *C. exul* show fine-scale preferences for some areas within the watercourse where reproductive activity is mostly concentrated. Such information is critical for establishing conservation strategies that increase the resilience of this endangered species to climate change and anthropogenic stress^31^.

Our results did not align with the hypothesis that there were survival costs related to movement. There have been only a few field CMR studies that showed survival costs related to movement in odonates^21^. Usually, movement has fitness costs^4,52^ that are related to not only energy expenditure but also predation risk. Species of Calopterygidae are often preyed on by birds, frogs, and spiders^53–55^. The absence of difference in survival between residents and movers in our studied population of *C. exul* was probably due to the low frequency of long-distance movements (> 100 m) which are typically costly^21^. In fact, the survival costs of movement observed in *C. splendens*^21^ was likely because of the more frequent longer movements (> 100 m). It is also likely that the costs related to movement are typically underestimated in CMR because transition data are confined to those individuals that we recaptured^21^. If so, the survival rate for *C. exul* movers could be overestimated^21^. Furthermore, although emigration out of the study area is possible, it is quite unlikely for *C. exul* because our results on movement probability exceeding 1 km (< 1%) were very rare and the nearest reproduction sites are a few kilometres away.

Furthermore, we found that females had higher survival rates than males. This finding is similar to that reported in other field studies on damselflies^16,56,57^ (albeit a general sex bias is not found in meta-analysis^58^) and other insect taxa^59,60^. For instance, in *Coenagrion puella* Linnaeus, Sherratt, et al.^61^ showed that female survival had a higher survival rate than males regardless of age. In *Ischnura elegans* Vander Linden, the daily probability of surviving was substantially higher in males (0.812) than in females (0.579)^62^. In neotropical damselflies (*Polythore mutata* McLachlan and *P. derivate* McLachlan), some populations showed a higher survival rate in females than males^56^. The observed difference in survival rates in *C. exul* could be due to the sexual difference in coloration and behavior, where males, being less cryptic and more active in chasing rival conspecifics, are more likely to die from predation.

Some models predicted that the survival probability of both males and females was lower when individuals were observed in areas of high male density. This is probably due to mating-related intraspecific interactions^63^ where male-male interference competition and female coercion incur damages and fitness costs^64^. Our results on density-dependent movement probability and distance are in line with this hypothesis, which has been suggested for many territorial species^65,66^, including invertebrates such as damselflies (*C. splendens*)^21^, butterflies^68^, flies^69^, backswimmers^70^, and vertebrates like birds and mammals^5^. Furthermore, the absence of sex-biased movement was less likely due to the low population size because our estimates of population size were large for the species^37^ (although the species no longer exists at the studied site as of 2023^27^, the population lived in large numbers in 2010 and 2011^18,37^). The lack of frequent movements could be due to preferences of particular sections of the river (philopatry), or the scarcity of high-quality reproductive habitats. Further investigations are needed to understand the movement behavior of the species and the potential occurrence of movement limitation.

### Conservation implications

Understanding the movement behavior of species is critical for managing natural populations and maintaining the long-term survival of species of conservation concern. Our study suggests that although *C. exul* is capable of performing movements that exceed 1 km, most of its movements are short distances (< 100 m) within certain patches where they forage and mate. Since our study was limited to the first cohort (spring–summer) of the species, we still need studies that compare the movement behavior of individuals during the first and second (autumn) cohorts to gain a better understanding of potential shifts in the species’ behavior, as was reported in other insect species^73^. Unfortunately, our understanding of the geographic distribution of the species in the Seybouse watershed suggests that the distances between the different sites where the species (used to) exist far exceed those usually travelled by the species in this study. The rapid recent retraction of the species distribution in the Seybouse watershed^27^ aligns with the hypothesis that the species is relatively dispersal-limited and has a low tolerance to declines in habitat quality. It seems that the continuous anthropogenic degradation of environmental conditions associated with severe drought is threatening the persistence of the species in the region^31^. Conservation plans should focus on improving environmental conditions and designing networks of high-quality habitat patches where anthropogenic disturbance is limited. This approach would ensure the persistence of the species. However, this remains challenging due to the increasing agricultural pressure on lotic waters through water extraction for irrigation, construction of dams, deviation of watercourses, and pollution by fertilizers and pesticides.

### Supplementary Information

Supplementary Information.

## Data Availability

Data are deposited in Figshare: https://figshare.com/s/2ccbabdd939a3cd6dbf3.
