# Supplementary material for: Condition-dependent survival and movement behavior in an endangered endemic damselfly
Source: Sci Rep. 2023 Dec 9;13:21819. doi: 10.1038/s41598-023-48162-w (PMC10710475; doi:10.1038/s41598-023-48162-w)
Supplement: Supplementary file 1 — Supplementary Information. [file 41598_2023_48162_MOESM1_ESM.docx]

**Supplementary materials**

**Condition-dependent survival and movement behavior in an endangered endemic damselfly**

Hayat Mahdjoub^1^, Rabah Zebsa^2^, Amin Kahalerras^3^, Hichem Amari^4^, Soufyane Bensouilah^5^, Michael Samways^6^, Rassim Khelifa^1,7*^

^1^ Biology Department, Concordia University, 7141 Sherbrooke St. W., Montreal, QC H4B 1R6, Canada

^2^ Department of Nature and Life Sciences, Faculty of Nature and Life Sciences and Earth and Universe Sciences, University of 08 May 1945, Guelma, Algeria

^3^ Direction Générale des Forêts, Guelma 24000, Algeria

^4^ Department of Natural Sciences, Ecole normale supérieure de Ouargla, Ouargla, Algeria

^5^ Biology Department, Université de Laghouat, Laghouat, Algeria

^6^ Department of Conservation Ecology and Entomology, Stellenbosch University, South Africa

^7^ Institute for Resources, Environment, and Sustainability, University of British Columbia, 2202 Main Mall, Vancouver, BC V6T 1Z4, Canada

Corresponding author (*): Rassim Khelifa [rassim.khelifa@concordia.ca](mailto:rassim.khelifa@concordia.ca)

**Table S1.** Physical characteristics of the sampling sections of the watercourse

| Section | Length (m) | Water width (m) | Water depth (cm) | Water velocity (m.s^-1^) | Bank vegetation height (cm) | Bank vegetation density (%) | Emergent stone density (%) | N |
| --- | --- | --- | --- | --- | --- | --- | --- | --- |
| A | 120 | 13.50 ± 3.37 | 102.92 ± 38.28 | 1.68 ± 0.36 | 173.75 ± 69.19 | 53.33 ± 26.31 | 3.75 ± 8.82 | 10 |
| B | 85 | 10.56 ± 3.32 | 84.44 ± 20.07 | 1.43 ± 0.40 | 180.56 ± 54.40 | 57.22 ± 19.22 | 0 | 10 |
| C | 130 | 15.40 ± 0.97 | 116.5 ± 25.17 | 0.29 ± 0.12 | 181.00 ± 14.49 | 67.50 ± 14.58 | 0 | 10 |
| E | 180 | 11.58 ± 2.91 | 107.5 ± 31.87 | 0.25 ± 0.11 | 157.50 ± 12.15 | 49.58 ± 24.16 | 1.67 ± 5.77 | 10 |
| F | 150 | 12 ± 2.53 | 72 ± 2.53 | 0.46 ± 0.01 | 193 ± 9.78 | 86.66 ± 5.23 | 0 | 10 |
| G | 140 | 12.50 ± 1.17 | 50.42 ± 6.56 | 1.29 ± 0.11 | 186.25 ± 69.71 | 59.58 ± 21.47 | 8.75 ± 13.51 | 10 |
| H | 90 | 25.20 ± 4.02 | 60 ± 11.18 | 60 ± 11.18 | 0.55 ± 0.03 | 108 ± 4.47 | 66 ± 2.23 | 10 |
| K | 140 | 10.77 ± 2.07 | 81.92 ± 21.26 | 0.97 ± 0.21 | 141.92 ± 37.05 | 39.62 ± 13.30 | 0.77 ± 2.77 | 10 |
| M | 80 | 15.43 ± 0.79 | 132.86 ± 10.75 | 0.21 ± 0.02 | 170.71 ± 9.32 | 69.29 ± 7.32 | 0 | 10 |
| P | 235 | 2.81 ± 1.50 | 39.04 ± 23.05 | 0.40 ± 0.22 | 217.39 ± 83.31 | 73.26 ± 16.14 | 2.39 ± 8.77 | 10 |

N: sample size

**Table S2.** Goodness of fit of the Multistate (MS) model for capture-mark-recapture data of *Calopteryx exul* in the Seybouse river for three distance thresholds of state transition (50 m, 100 m, and 500 m). The non-significance of all tests shows that there is no departure from the MS model assumptions.

| Distance threshold (m) | Test | Chi.square | df | P |
| --- | --- | --- | --- | --- |
| 50 | TEST2 | 55.6 | 53 | 0.377 |
| 50 | TEST3 | 50.7 | 73 | 0.979 |
| 50 | Total | 106.3 | 126 | 0.898 |
|  |  |  |  |  |
| 100 | TEST2 | 56.1 | 57 | 0.508 |
| 100 | TEST3 | 49.0 | 73 | 0.986 |
| 100 | Total | 105.1 | 130 | 0.947 |
|  |  |  |  |  |
| 500 | TEST2 | 75.9 | 59 | 0.069 |
| 500 | TEST3 | 52.3 | 73 | 0.968 |
| 500 | Total | 128.1 | 132 | 0.579 |

TEST2 assesses trap-dependence (whether captured individuals have equal recapture probabilities); TEST3 assesses transience (whether newly marked insects at occasion t have less chance to be recaptured at t + 1 than insects already marked at t).

**Table S3.** Model selection of the Multistate models of detection (p), survival (S), and transition (Psi) probabilities of *Calopteryx exul* in the Seybouse river using a distance threshold of state transition of 50 m. Here, individuals that moved more than 50 m from their previous positions were considered movers. The procedure of model selection was conducted such that S is fixed as constant whereas Psi depends on state. Then, the models of p varied using biologically meaningful models that include gradual complexity of covariates. After selecting the best (parsimonious models with ΔAICc ≤ 2) model for p, we fix it, and vary models for S using the same procedure. The same analytical steps are repeated for Psi. Models are ranked based on AICc. sr: sex ratio; st: state. (.) means constant model.

|  | npar | AICc | ΔAICc | weight | Deviance |
| --- | --- | --- | --- | --- | --- |
| Recapture probability models |  |  |  |  |  |
| S(Sex + st + density)p(Sex + time + st)Psi(st) | 29 | 3856.7 | 0.000 | 0.257 | 3797.2 |
| S(Sex + st + density + sr + Time)p(Sex + time + st)Psi(st) | 31 | 3856.9 | 0.194 | 0.233 | 3793.1 |
| S(Sex)p(Sex + time + st)Psi(st) | 27 | 3857.4 | 0.756 | 0.176 | 1939.6 |
| S(Sex + st + density + sr)p(Sex + time + st)Psi(st) | 30 | 3857.4 | 0.760 | 0.176 | 3795.8 |
| S(Sex + st + density * sr + Time)p(Sex + time + st)Psi(st) | 32 | 3858.9 | 2.223 | 0.085 | 3793.0 |
| S(Sex + st)p(Sex + time + st)Psi(st) | 28 | 3859.2 | 2.526 | 0.073 | 1939.3 |
|  |  |  |  |  |  |
| Survival probability models |  |  |  |  |  |
| S(Sex + st + density)p(Sex + time + st)Psi(st) | 29 | 3856.7 | 0.000 | 0.257 | 3797.2 |
| S(Sex + st + density + sr + Time)p(Sex + time + st)Psi(st) | 31 | 3856.9 | 0.194 | 0.233 | 3793.1 |
| S(Sex)p(Sex + time + st)Psi(st) | 27 | 3857.4 | 0.756 | 0.176 | 1939.6 |
| S(Sex + st + density + sr)p(Sex + time + st)Psi(st) | 30 | 3857.4 | 0.760 | 0.176 | 3795.8 |
| S(Sex + st + density * sr + Time)p(Sex + time + st)Psi(st) | 32 | 3858.9 | 2.223 | 0.085 | 3793.0 |
| S(Sex + st)p(Sex + time + st)Psi(st) | 28 | 3859.2 | 2.526 | 0.073 | 1939.3 |
|  |  |  |  |  |  |
| Transition probability models |  |  |  |  |  |
| S(Sex)p(Sex + time + st)Psi(st + st:density) | 29 | 3850.9 | 0.000 | 0.731 | 3791.4 |
| S(Sex)p(Sex + time + st)Psi(st + st:density + st:sr) | 31 | 3853.6 | 2.754 | 0.184 | 3789.9 |
| S(Sex)p(Sex + time + st)Psi(st) | 27 | 3857.4 | 6.521 | 0.028 | 1939.6 |
| S(Sex)p(Sex + time + st)Psi(st + st:Sex + st:density + st:sr) | 33 | 3857.7 | 6.856 | 0.024 | 3789.8 |
| S(Sex)p(Sex + time + st)Psi(.) | 26 | 3858.0 | 7.075 | 0.021 | 1942.2 |
| S(Sex)p(Sex + time + st)Psi(st + st:sr) | 29 | 3860.3 | 9.432 | 0.007 | 3800.8 |
| S(Sex)p(Sex + time + st)Psi(st + st:Sex) | 29 | 3860.6 | 9.735 | 0.006 | 1938.6 |

**Table S4.** Model selection of the Multistate models of detection (p), survival (S), and transition (Psi) probabilities of *Calopteryx exul* in the Seybouse river using a distance threshold of state transition of 100 m. Here, individuals that moved more than 100 m from their previous positions were considered movers. The procedure of model selection was conducted such that S is fixed as constant whereas Psi depends on state. Then, the models of p varied using biologically meaningful models that include gradual complexity of covariates. After selecting the best (parsimonious models with ΔAICc ≤ 2) model for p, we fix it, and vary models for S using the same procedure. The same analytical steps are repeated for Psi. Models are ranked based on AICc. sr: sex ratio; st: state. (.) means constant model.

|  | npar | AICc | ΔAICc | weight | Deviance |
| --- | --- | --- | --- | --- | --- |
| Recapture probability models |  |  |  |  |  |
| S(.)p(Sex + time + st)Psi(st) | 26 | 3735.3 | 0.0 | 1.000 | 1822.3 |
| S(.)p(Sex + st)Psi(st) | 6 | 3757.4 | 22.1 | 0.000 | 1885.6 |
| S(.)p(Sex + time)Psi(st) | 25 | 3769.5 | 34.2 | 0.000 | 1858.6 |
| S(.)p(time)Psi(st) | 24 | 3775.2 | 39.9 | 0.000 | 1866.5 |
| S(.)p(Sex)Psi(st) | 5 | 3796.1 | 60.9 | 0.000 | 1926.3 |
| S(.)p(.)Psi(st) | 4 | 3799.5 | 64.2 | 0.000 | 1931.7 |
|  |  |  |  |  |  |
| Survival probability models |  |  |  |  |  |
| S(Sex + st + density)p(Sex + time + st)Psi(st) | 29 | 3733.3 | 0.0 | 0.292 | 3673.8 |
| S(Sex + st + density + sr)p(Sex + time + st)Psi(st) | 30 | 3734.1 | 0.8 | 0.196 | 3672.5 |
| S(Sex)p(Sex + time + st)Psi(st) | 27 | 3734.1 | 0.8 | 0.193 | 1819.1 |
| S(Sex + st + density + sr + Time)p(Sex + time + st)Psi(st) | 31 | 3734.3 | 0.9 | 0.183 | 3670.5 |
| S(Sex + st)p(Sex + time + st)Psi(st) | 28 | 3736.2 | 2.9 | 0.069 | 1819.1 |
| S(Sex + st + density * sr + Time)p(Sex + time + st)Psi(st) | 32 | 3736.2 | 2.9 | 0.068 | 3670.4 |
|  |  |  |  |  |  |
| Transition probability models |  |  |  |  |  |
| S(Sex)p(Sex + time + st)Psi(st + st:density) | 29 | 3727.5 | 0.0 | 0.657 | 3668.0 |
| S(Sex)p(Sex + time + st)Psi(st + st:density + st:sr) | 31 | 3729.7 | 2.2 | 0.218 | 3666.0 |
| S(Sex)p(Sex + time + st)Psi(.) | 26 | 3732.3 | 4.8 | 0.061 | 1819.3 |
| S(Sex)p(Sex + time + st)Psi(st + st:Sex + st:density + st:sr) | 33 | 3733.9 | 6.4 | 0.027 | 3665.9 |
| S(Sex)p(Sex + time + st)Psi(st) | 27 | 3734.1 | 6.6 | 0.024 | 1819.1 |
| S(Sex)p(Sex + time + st)Psi(st + st:sr) | 29 | 3736.6 | 9.1 | 0.007 | 3677.1 |
| S(Sex)p(Sex + time + st)Psi(st + st:Sex) | 29 | 3737.0 | 9.5 | 0.006 | 1817.8 |

**Table S5.** Model selection of the Multistate models of detection (p), survival (S), and transition (Psi) probabilities of *Calopteryx exul* in the Seybouse river using a distance threshold of state transition of 500 m. Here, individuals that moved more than 500 m from their previous positions were considered movers. The procedure of model selection was conducted such that S is fixed as constant whereas Psi depends on state. Then, the models of p varied using biologically meaningful models that include gradual complexity of covariates. After selecting the best (parsimonious models with ΔAICc ≤ 2) model for p, we fix it, and vary models for S using the same procedure. The same analytical steps are repeated for Psi. Models are ranked based on AICc. sr: sex ratio; st: state. (.) means constant model.

|  | npar | AICc | ΔAICc | weight | Deviance |
| --- | --- | --- | --- | --- | --- |
| Recapture probability models |  |  |  |  |  |
| S(.)p(Sex + time + st)Psi(st) | 26 | 3401.7 | 0.000 | 1.000 | 1537.5 |
| S(.)p(Sex + st)Psi(st) | 6 | 3417.8 | 16.095 | 0.000 | 1594.7 |
| S(.)p(Sex + time)Psi(st) | 25 | 3425.1 | 23.389 | 0.000 | 1562.9 |
| S(.)p(time)Psi(st) | 24 | 3430.9 | 29.132 | 0.000 | 1570.8 |
| S(.)p(Sex)Psi(st) | 5 | 3451.8 | 50.033 | 0.000 | 1630.7 |
| S(.)p(.)Psi(st) | 4 | 3455.1 | 53.414 | 0.000 | 1636.0 |
| S(.)p(.)Psi(.) | 3 | 3474.5 | 72.725 | 0.000 | 1657.4 |
|  |  |  |  |  |  |
| Survival probability models |  |  |  |  |  |
| S(Sex + st + density + sr + Time)p(Sex + time + st)Psi(st) | 31 | 3398.7 | 0.000 | 0.474 | 3335.0 |
| S(Sex + st + density + sr)p(Sex + time + st)Psi(st) | 30 | 3400.7 | 1.972 | 0.177 | 3339.1 |
| S(Sex)p(Sex + time + st)Psi(st) | 27 | 3400.7 | 1.979 | 0.176 | 1534.3 |
| S(Sex + st + density * sr + Time)p(Sex + time + st)Psi(st) | 32 | 3400.7 | 2.005 | 0.174 | 3334.9 |
| S(Sex + st + density)p(Sex + time + st)Psi(st) | 29 | 3433.2 | 34.526 | 0.000 | 3373.7 |
| S(Sex + st)p(Sex + time + st)Psi(st) | 28 | 3435.0 | 36.237 | 0.000 | 1566.5 |
|  |  |  |  |  |  |
| Transition probability models |  |  |  |  |  |
| S(Sex)p(Sex + time + st)Psi(st + st:density) | 29 | 3397.6 | 0.000 | 0.515 | 3338.1 |
| S(Sex)p(Sex + time + st)Psi(.) | 26 | 3399.6 | 1.906 | 0.199 | 1535.3 |
| S(Sex)p(Sex + time + st)Psi(st + st:density + st:sr) | 31 | 3400.5 | 2.877 | 0.122 | 3336.8 |
| S(Sex)p(Sex + time + st)Psi(st) | 27 | 3400.7 | 3.055 | 0.112 | 1534.3 |
| S(Sex)p(Sex + time + st)Psi(st + st:Sex) | 29 | 3403.2 | 5.566 | 0.032 | 1532.6 |
| S(Sex)p(Sex + time + st)Psi(st + st:sr) | 29 | 3404.1 | 6.499 | 0.020 | 3344.6 |
